# A Cyanobacterial Component Required for Pilus Biogenesis Affects the Exoproteome

**DOI:** 10.1128/mBio.03674-20

**Published:** 2021-03-16

**Authors:** Yevgeni Yegorov, Eleonora Sendersky, Shaul Zilberman, Elad Nagar, Hiba Waldman Ben-Asher, Eyal Shimoni, Ryan Simkovsky, Susan S. Golden, Andy LiWang, Rakefet Schwarz

**Affiliations:** a The Mina and Everard Goodman Faculty of Life Sciences, Bar-Ilan University, Ramat-Gan, Israel; b Department of Chemical Research Support, Weizmann Institute of Science, Rehovot, Israel; c Division of Biological Sciences, University of California, San Diego, La Jolla, California, USA; d Center for Circadian Biology, University of California, San Diego, La Jolla, California, USA; e Department of Chemistry and Chemical Biology, University of California, Merced, Merced, California, USA; f Center for Cellular and Biomolecular Machines, University of California, Merced, Merced, California, USA; g Health Sciences Research Institute, University of California, Merced, Merced, California, USA; University of Washington

**Keywords:** *Synechococcus elongatus*, biofilms, cyanobacteria, pilus assembly, protein secretion

## Abstract

Protein secretion as well as the assembly of bacterial motility appendages are central processes that substantially contribute to fitness and survival. This study highlights distinctive features of the mechanism that serves these functions in cyanobacteria, which are globally prevalent photosynthetic prokaryotes that significantly contribute to primary production. Our studies of biofilm development in the cyanobacterium Synechococcus elongatus uncovered a novel component required for the biofilm self-suppression mechanism that operates in this organism. This protein, which is annotated as “hypothetical,” is denoted EbsA (essential for biofilm self-suppression A) here. EbsA homologs are highly conserved and widespread in diverse cyanobacteria but are not found outside this clade. We revealed a tripartite complex of EbsA, Hfq, and the ATPase homolog PilB (formerly called T2SE) and demonstrated that each of these components is required for the assembly of the hairlike type IV pili (T4P) appendages, for DNA competence, and affects the exoproteome in addition to its role in biofilm self-suppression. These data are consistent with bioinformatics analyses that reveal only a single set of genes in *S. elongatus* to serve pilus assembly or protein secretion; we suggest that a single complex is involved in both processes. A phenotype resulting from the impairment of the EbsA homolog in the cyanobacterium *Synechocystis* sp. strain PCC 6803 implies that this feature is a general cyanobacterial trait. Moreover, comparative exoproteome analyses of wild-type and mutant strains of *S. elongatus* suggest that EbsA and Hfq affect the exoproteome via a process that is independent of PilB, in addition to their involvement in a T4P/secretion machinery.

## Abstract (precis)

Cyanobacteria, environmentally prevalent photosynthetic prokaryotes, contribute ∼25% of global primary production. Cyanobacterial biofilms elicit biofouling, thus leading to substantial economic losses; however, these microbial assemblages can also be beneficial, e.g., in wastewater purification processes and for biofuel production.

## INTRODUCTION

Protein secretion is a fundamental cellular process that plays a pivotal role in all living cells. Bacteria evolved various secretion complexes that are classified based on subunit composition, structural similarity, and cellular role (1, 2). These nanomachines include type I systems, relatively simple complexes commonly consisting of three proteins that mediate a single-step secretion process in which substrates are transported from the cytoplasm to the extracellular milieu (3, 4). Type II secretion (T2S) systems, much larger complexes that comprise at least 12 different components, secrete periplasmically located proteins across the outer membrane (5–8). These complexes, which take part in a two-step secretion process, depend on transport across the inner membrane to the periplasm by Sec or Tat machineries (9). T2S systems are evolutionarily related to type IV pilus assembly (T4P) systems, and components of these complexes exhibit sequence and structural similarities (10). Additionally, many bacteria have evolved specialized complexes that enable the injection of bacterially encoded effector proteins into eukaryotic cells (11–16). These secretion machineries, termed type III (T3S), type IV (T4S), and type VI (T6S) secretion systems, play a central role in pathogenic or symbiotic interactions (17). Intriguingly, a recent study demonstrated that enteropathogenic Escherichia coli utilizes components of a T3S system as a platform for nanotube-mediated extraction of host nutrients (18).

Cyanobacteria are widespread in nature and contribute substantially to global primary productivity (19). Protein secretion in these photosynthetic prokaryotes is understudied, and much less is known than the vast knowledge of these mechanisms in heterotrophic bacteria. Several cyanobacterial type I-like systems have been documented (20); e.g., TolC-like proteins were demonstrated to be involved in protein secretion in *Anabaena* sp. strain PCC 7120 (21) and *Synechocystis* (22). Additional ATP-binding cassette transports were characterized in *Anabaena* (23).

Cyanobacteria are often found as part of microbial assemblages known as biofilms (24, 25). Despite their environmental prevalence and the industrial problems associated with phototrophic biofilms (26, 27), the mechanisms that underlie cyanobacterial biofilm development have only begun to emerge in recent years. For example, cyclic di-GMP, a known second messenger that regulates biofilm development in heterotrophic bacteria, is also involved in cyanobacterial biofilm formation (28, 29).

Our previous studies of the unicellular model cyanobacterium Synechococcus elongatus revealed that the constitutive expression of a biofilm self-suppression mechanism is responsible for the persistent planktonic growth of the wild-type (WT) (PCC 7942) strain under standard laboratory conditions (30, 31). The ability to develop robust biofilms is recovered in this laboratory strain when the Synpcc7942_2071 gene (annotated as ATPase) is inactivated. Imaging by scanning electron microscopy of this biofilm-forming mutant revealed multilayer biofilms and extracellular matrix (30, 32). Prior studies show that the extracellular fluid (referred to here as conditioned medium [CM]) from the mutant lacks a biofilm-suppressing factor that is present in CM from the WT (30). Moreover, analysis of CM by electrophoresis and silver staining as well as by mass spectrometry (MS) indicated that the inactivation of this gene impairs protein secretion (33). The protein encoded by Synpcc7942_2071 is characterized by the conserved domains T2SSE_N (T2S system subunit E [T2SSE] N-terminus) and T2SSE; thus, it was previously designated T2SE (30). Additionally, the mutant lacks cell pili (33), in accordance with the presence of T2SSE_N and T2SSE domains in assembly ATPases of T4P systems. Data mining predicted very few T2S systems outside *Proteobacteria*, and it was suggested that in their canonical form, these secretion systems are limited to this phylum (7, 34). Given this bioinformatic information along with the observation that the inactivation of Synpcc7942_2071 abrogates pilus formation, we rename this gene product PilB and the corresponding mutant *pilB*::Tn*5*. Here, we provide further evidence suggesting the involvement of the T4P machinery of S. elongatus in protein secretion, as previously shown for several heterotrophic bacteria (35), and identify a component of the complex that is conserved specifically in the cyanobacterial clade.

Robust biofilm development is observed when the *pilB*::Tn*5* mutant is grown in fresh medium; however, when inoculated into CM from a WT culture, the mutant grows planktonically. CM supplemented with nutrients still suppresses biofilm formation, negating the possibility that nutrient starvation prevented biofilm formation. Furthermore, suppressive fractions identified from the CM implicate a small (<3 kDa) heat-resistant substance as the inhibitor (30). The inhibitory effect of WT CM on biofilm development by the *pilB*::Tn*5* mutant is in accordance with a model that assigns a role for the *S. elongatus* T4P machinery in the deposition of a biofilm inhibitor to the extracellular milieu.

Components that promote biofilm development in *S. elongatus* include four small proteins, EbfG1 (enable biofilm formation with a GG motif 1) to EbfG4, which are secreted by a mechanism employing PteB (32) (peptidase transporter essential for biofilm) and the microcin-processing peptidase-like protein EbfE (36). The extracellular biofilm inhibitor represses the transcription of the genes *ebfG1–4* and *pteB*, thereby prohibiting biofilm development (31, 32).

Here, we describe additional components that are essential for the biofilm self-suppression mechanism in *S. elongatus*. A genetic approach uncovered a highly conserved cyanobacterial protein (EbsA [essential for biofilm suppression A]) that does not exhibit sequence similarity to proteins of known function. Structurally, EbsA belongs to the α+β class and has a roll architecture; however, comparisons with other protein structures did not provide significant insights into its role in biofilm suppression (37). We revealed a tripartite complex of EbsA, PilB, and the Hfq RNA chaperone. Mutants defective for any one of these components develop biofilms and are impaired in protein secretion, pilus assembly, and competence for DNA uptake. Together, the data from this study reveal previously unrecognized components of pilus assembly, and possibly of a secretion system, thereby suggesting a novel mode of operation of these cyanobacterial complexes.

## RESULTS

### Synpcc7942_0862 encodes a highly conserved cyanobacterial protein required for biofilm self-suppression.

**FIG 1:**
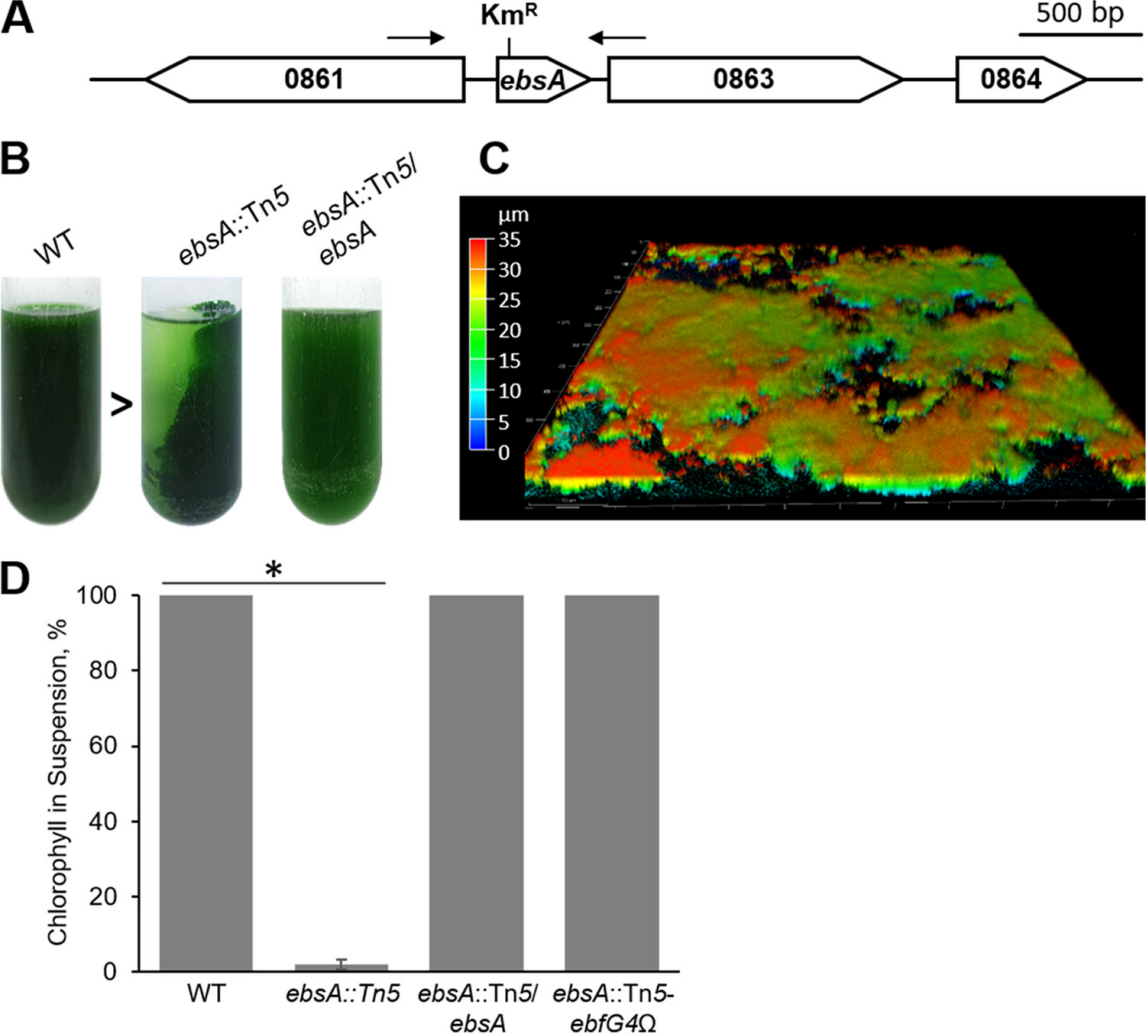
Synpcc7942_0862 is required for biofilm self-suppression. (A) Map of the genomic region of Synpcc7942_0862. All indicated genes are annotated as “conserved hypothetical proteins.” Unigene Set (UGS) vector 22D7 was used to insert Tn*5* encoding a kanamycin resistance cassette (Km^R^) to generate the *ebsA*::Tn*5* gene inactivation mutant. Arrows indicate PCR primers used to amplify a DNA fragment that was introduced in *trans* into *ebsA*::Tn*5* to generate the *ebsA*::Tn*5*/*ebsA* strain to test complementation (see Table S1 in the supplemental material for details). (B) Cultures of WT, *ebsA*::Tn*5*, and *ebsA*::Tn*5*/*ebsA* strains. The arrowhead indicates the direction of light (biofilms are formed away from the light source). (C) Image of *ebsA*::Tn*5* biofilm using confocal fluorescence microscopy. Imaging is based on autofluorescence (excitation at 630 nm and emission at 641 to 657 nm). The color scale represents biofilm depth. Different regions of the biofilm differ in depth (Fig. S2). (D) Percentage of chlorophyll in suspension as a proxy for biofilm formation. Strains analyzed were the WT, the *ebsA*::Tn*5* mutant, the *ebsA*::Tn*5*/*ebsA* strain, and the double mutant in which *ebsA* and gene Synpcc7942_1134 are inactivated (*ebsA*::Tn*5 ebfG4*). Data represent averages and standard deviations (SD) from 3 independent experiments. The asterisk indicates significance (*P* < 5.7E−30 by a two-tailed *t* test).

The biofilm-forming *pilB*::Tn*5* mutant is unpiliated and characterized by a high rate of sedimentation compared to the WT (33). This correlation between fast cell sedimentation and biofilm development in the *pilB*::Tn*5* mutant encouraged us to test additional rapidly sedimenting mutants for biofilm development. Four of the 68 transposon insertion mutants that were classified as fast sedimenting in a genetic screen of an *S. elongatus* mutant library (38) exhibited biofilm formation (33). Three of these mutants bear an inactivation of genes related to T2S or T4P systems (*pilA2*, *pilC*, and *pilN*) (33). The fourth mutant is inactivated in Synpcc7942_0862 (Fig. 1A), dubbed *ebsA* (essential for biofilm suppression A) here. Homologs of EbsA that share 44 to 52% identity and 68 to 73% similarity (see Fig. S1 in the supplemental material) are widespread among diverse cyanobacteria but are not found outside this clade. In contrast to the WT strain that grows planktonically (Fig. 1B and Fig. S2), the Synpcc7942_0862 mutant (*ebsA*::Tn*5*) formed robust biofilms, as shown by adhesion to the growth vessel (Fig. 1B) and by confocal fluorescence microscopy (Fig. 1C and Fig. S2).

10.1128/mBio.03674-20.1 FIG S1 Sequence alignment of homologs of *S. elongatus* EbsA from diverse cyanobacteria, including freshwater, marine, unicellular, and filamentous species. Numbering is according to the EbsA sequence. Download FIG S1, TIF file, 3.0 MB. Copyright © 2021 Yegorov et al. 2021 Yegorov et al. https://creativecommons.org/licenses/by/4.0/ This content is distributed under the terms of the Creative Commons Attribution 4.0 International license.

10.1128/mBio.03674-20.2 FIG S2 Imaging of biofilms by confocal fluorescence microscopy. Imaging is based on autofluorescence (excitation at 630 nm and emission at 641 to 657 nm). The color scale represents biofilm depth. Strains analyzed were the *S. elongatus* WT, the *ebsA*::Tn*5* and *hfq*Ω mutants, and cognate complemented strains (*ebsA*::Tn*5*/*ebsA* and *hfq*Ω/*hfq*, respectively). Download FIG S2, TIF file, 3.0 MB. Copyright © 2021 Yegorov et al. 2021 Yegorov et al. https://creativecommons.org/licenses/by/4.0/ This content is distributed under the terms of the Creative Commons Attribution 4.0 International license.

10.1128/mBio.03674-20.6 TABLE S1 Summary of cloning information. *The gene inactivation vector was initially constructed by cloning a PCR product amplified with the indicated primers into pJET1.2. Subsequently, the specified restriction site was used for the insertion of either a chloramphenicol resistance cassette (Cm^R^) that is based on the Ω inactivation fragment [P. Prentki and H. M. Krisch, Gene 29:303–313, 1984, https://doi.org/10.1016/0378-1119(84)90059-3] or a kanamycin resistance cassette (Km^R^) from the transposon Tn*5* [E. Beck, G. Ludwig, E. A. Auerswald, B. Reiss, and H. Schaller, Gene 19:327–336, 1982, https://doi.org/10.1016/0378-1119(82)90023-3], as indicated. Accordingly, the mutants are denoted *hfq*Ω and *ebsA*::Tn*5*. **Transposon insertional inactivation was performed using the indicated vector from the Unigene Set (UGS) library (C. K. Holtman, Y. Chen, P. Sandoval, A. Gonzales, et al., DNA Res 12:103–115, 2005, https://doi.org/10.1093/dnares/12.2.103). Gene disruption in *S. elongatus* was obtained by transformation and replacement of the native gene by homologous recombination. #PCR products were cloned into SwaI in NSII or NSI (*hfq* and *pilB*, respectively) or EcoRV in the shuttle vector pSZ2 (*ebsA*). All cloning products were validated by PCR analyses and sequencing. Complete chromosome segregation of cyanobacterial strains was confirmed by PCR. Lowercase letters in primers indicate the regions that encode the triple-FLAG tag. Download Table S1, PDF file, 0.8 MB. Copyright © 2021 Yegorov et al. 2021 Yegorov et al. https://creativecommons.org/licenses/by/4.0/ This content is distributed under the terms of the Creative Commons Attribution 4.0 International license.

Additionally, measurement of the relative amount of chlorophyll in the planktonic fraction of the culture as a proxy for biofilm development (39) demonstrated that less than 10% is present in suspended cells of the mutant (Fig. 1D, *ebsA*::Tn*5*), indicating that the majority of the cells were in the sessile fraction. The introduction, in *trans*, of an intact copy of the *ebsA* gene preceded by an upstream sequence, presumed to include its promoter, restored planktonic growth to the *ebsA*::Tn*5* mutant (Fig. 1A and D and Fig. S2, *ebsA*::Tn*5*/*ebsA*), validating that biofilm formation is due to the inactivation of this gene. The inactivation of *ebsA* did not affect growth as measured by the increase in cell density at the planktonic stage of the cultures (Fig. S3A) and by total chlorophyll following biofilm formation (Fig. S3B). Thus, it is unlikely that biofilm development by this mutant is the consequence of a stress response elicited by the inactivation of *ebsA*. Together, the data support the involvement of *ebsA* in the biofilm self-suppression mechanism that operates in *S. elongatus*.

10.1128/mBio.03674-20.3 FIG S3 Inactivation of either *ebsA* or *hfq* does not affect growth. (A) Optical density at 750 nm (OD_750_) as a function of time in WT, *pilB*::Tn*5*, *ebsA*::Tn*5*, and *hfq*Ω cultures. Cultures were monitored for up to 3 days (the planktonic stage of the mutant cultures). Averages ± SD from 3 technical repeats from a representative experiment are shown. (B) Total chlorophyll in 7-day-old cultures. Averages ± SD from 3 independent experiments are shown. Download FIG S3, TIF file, 3.0 MB. Copyright © 2021 Yegorov et al. 2021 Yegorov et al. https://creativecommons.org/licenses/by/4.0/ This content is distributed under the terms of the Creative Commons Attribution 4.0 International license.

Previously, we demonstrated that the double mutant in which both *pilB* and Synpcc7942_1134 (encoding EbfG4) are insertionally inactivated grows planktonically, in contrast to the biofilm-forming *pilB* mutant (30). Likewise, the *ebsA*::Tn*5 ebfG4*Ω double mutant grew planktonically, in contrast to the biofilm-forming phenotype of the *ebsA*::Tn*5* mutant (Fig. 1D). These observations suggest that both the *pilB*::Tn*5* and *ebsA*::Tn*5* mutants employ a mechanism of biofilm development that requires EbfG4.

### Coimmunoprecipitation reveals a tripartite complex of EbsA, PilB, and the RNA chaperone Hfq.

**FIG 2:**
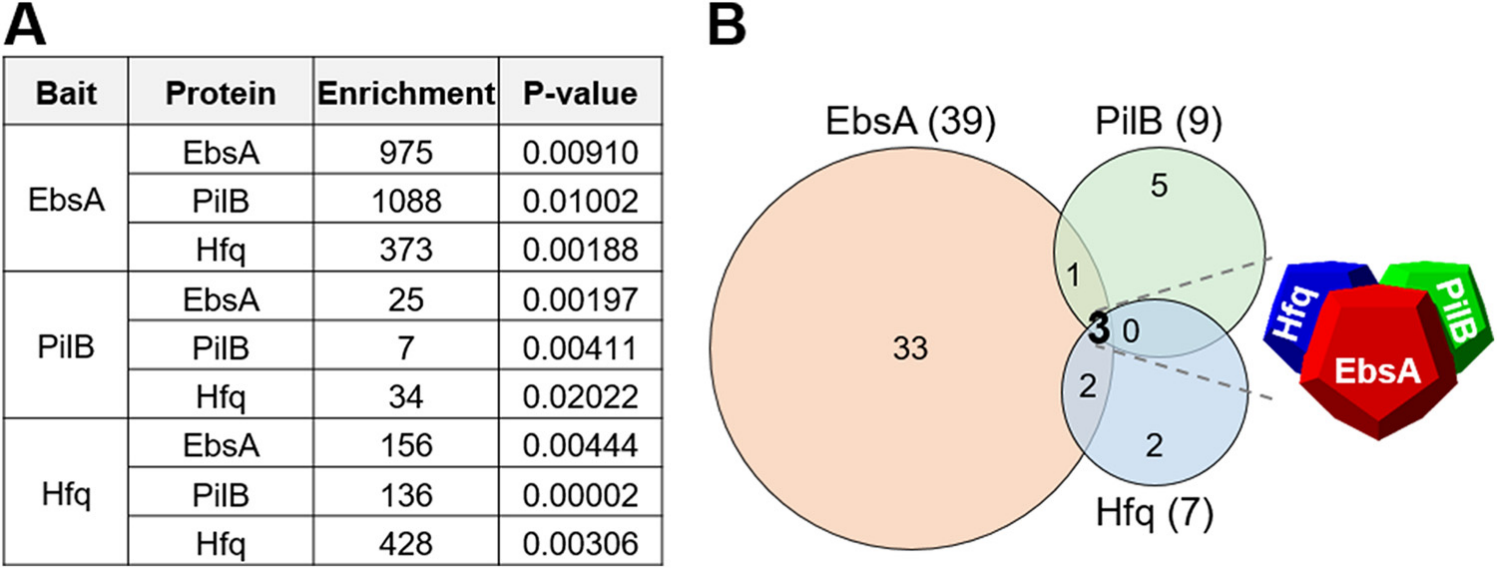
Immunoprecipitation (IP) followed by MS analysis. (A) Data for proteins immunoprecipitated using FLAG-tagged EbsA, PilB, or Hfq bait proteins. “Enrichment” indicates the ratio of protein detected in FLAG-tagged versus nontagged samples. Data are from three independent biological replicates. The *P* value from Student’s *t* test is indicated. Standard deviations are included in Data Set S1 in the supplemental material. (B) Venn diagrams summarizing co-IP experiments. The numbers of proteins specifically enriched following precipitation with a particular bait (fold change of >6; *P* value of <0.05) are indicated in parentheses. Numbers in overlapping areas indicate proteins common to the indicated IP baits. The three proteins common to all co-IP experiments (EbsA, PilB, and Hfq [boldface type]) form a tripartite complex.

To gain insight into EbsA function, we aimed at the identification of its interactors by coimmunoprecipitation (co-IP). To this end, the protein was tagged at its C terminus with a triple-FLAG epitope tag (here, EbsA::FLAG). The expression of the gene that encodes this tagged protein under the control of its native regulatory region complemented the biofilm-forming phenotype of the *ebsA*-inactivated strain (Fig. S4A, *ebsA*::Tn*5*/*ebsA*::flag). Cell extracts from the *ebsA*::Tn*5*/*ebsA*::flag and *ebsA*::Tn*5*/*ebsA* strains were used for co-IP experiments followed by MS analysis. Protein enrichment was calculated as the amount of protein in the “bait strain” (*ebsA*::Tn*5*/*ebsA*::flag) relative to its cognate control (*ebsA*::Tn*5*/*ebsA*). These experiments indicated high enrichment of PilB (Fig. 2A), the ATPase homolog required for the biofilm self-inhibitory mechanism. This observation, which indicates a physical interaction between EbsA and PilB, is consistent with a biofilm-inhibitory role for EbsA, as evidenced by *ebsA*::Tn*5* biofilm development.

10.1128/mBio.03674-20.4 FIG S4 Phenotypic complementation of the *ebsA*::Tn*5*, *pilB*::Tn*5*, and *hfq*Ω mutants with cognate genes encoding FLAG-tagged proteins. Percentages of chlorophyll in suspension as a proxy for biofilm formation are shown. Download FIG S4, TIF file, 2.1 MB. Copyright © 2021 Yegorov et al. 2021 Yegorov et al. https://creativecommons.org/licenses/by/4.0/ This content is distributed under the terms of the Creative Commons Attribution 4.0 International license.

10.1128/mBio.03674-20.8 DATA SET S1 Coimmunoprecipitation (co-IP) summary: proteins significantly enriched in IPs. Enrichment and Student’s *t* test *P* values are shown for proteins with significant enrichment (enrichment of >6; *P* value of <0.05). Data for all detected proteins from each IP are supplied in the particular data sheets. Download Data Set S1, XLSX file, 0.3 MB. Copyright © 2021 Yegorov et al. 2021 Yegorov et al. https://creativecommons.org/licenses/by/4.0/ This content is distributed under the terms of the Creative Commons Attribution 4.0 International license.

To validate the interactions between EbsA and PilB, the latter was FLAG tagged (Table S1), and the tagged protein, PilB::FLAG, was demonstrated to complement the *pilB* mutant (Fig. S4B, *pilB*::Tn*5*/*pilB*::flag). Co-IP experiments using PilB::FLAG indicated coprecipitation of EbsA, further confirming a PilB-EbsA interaction (Fig. 2). We also noted that both EbsA and PilB interact with the protein encoded by Synpcc7942_2070, a homolog of the PilT ATPase of T4P systems (Data Set S1). These interactions suggest a role for EbsA in pilus assembly.

Intriguingly, a homolog of the RNA chaperone Hfq (encoded by Synpcc7942_1926) was also highly enriched by co-IP with both EbsA::FLAG and PilB::FLAG baits (Fig. 2). Hfq is a central bacterial regulator that acts at the posttranscriptional level by mediating mRNA-small RNA interactions (40–43). These co-IP data suggest a tripartite complex of EbsA, PilB, and Hfq. Indeed, EbsA and PilB were two of only seven highly enriched proteins when Hfq::FLAG was used as a bait for co-IP (Fig. 2A and Data Set S1), further substantiating the presence of the tripartite EbsA-PilB-Hfq complex.

Studies of *Synechocystis* demonstrated that Hfq interacts with PilB (44), suggesting that the association of this RNA chaperone with T4P machinery is a more general cyanobacterial trait. RNA substrates of cyanobacterial Hfq proteins, however, have not been identified (44).

Co-IP data revealed 39 proteins interacting with EbsA (fold change of >6; *P* value of <0.05) (Data Set S1), of which 33 did not interact with PilB or Hfq (Fig. 2B). Analysis for motifs using Multiple Em for Motif Elicitation (MEME) (45) did not reveal protein motifs that are significantly enriched in EbsA interactors. Analysis by STRING, however, indicated that molecular functions related to magnesium ion binding and protein domains related to different ATPase families are enriched among EbsA interactors (Data Set S2).

10.1128/mBio.03674-20.9 DATA SET S2 Summary of STRING analyses for EbsA interactors and for proteins that were significantly less abundant in exoproteomes of the *ebsA*::Tn*5*, *pilB*::Tn*5*, and *hfq*Ω mutants than in the WT exoproteome. Download Data Set S2, XLSX file, 0.02 MB. Copyright © 2021 Yegorov et al. 2021 Yegorov et al. https://creativecommons.org/licenses/by/4.0/ This content is distributed under the terms of the Creative Commons Attribution 4.0 International license.

### Hfq is essential for biofilm self-suppression.

**FIG 3:**
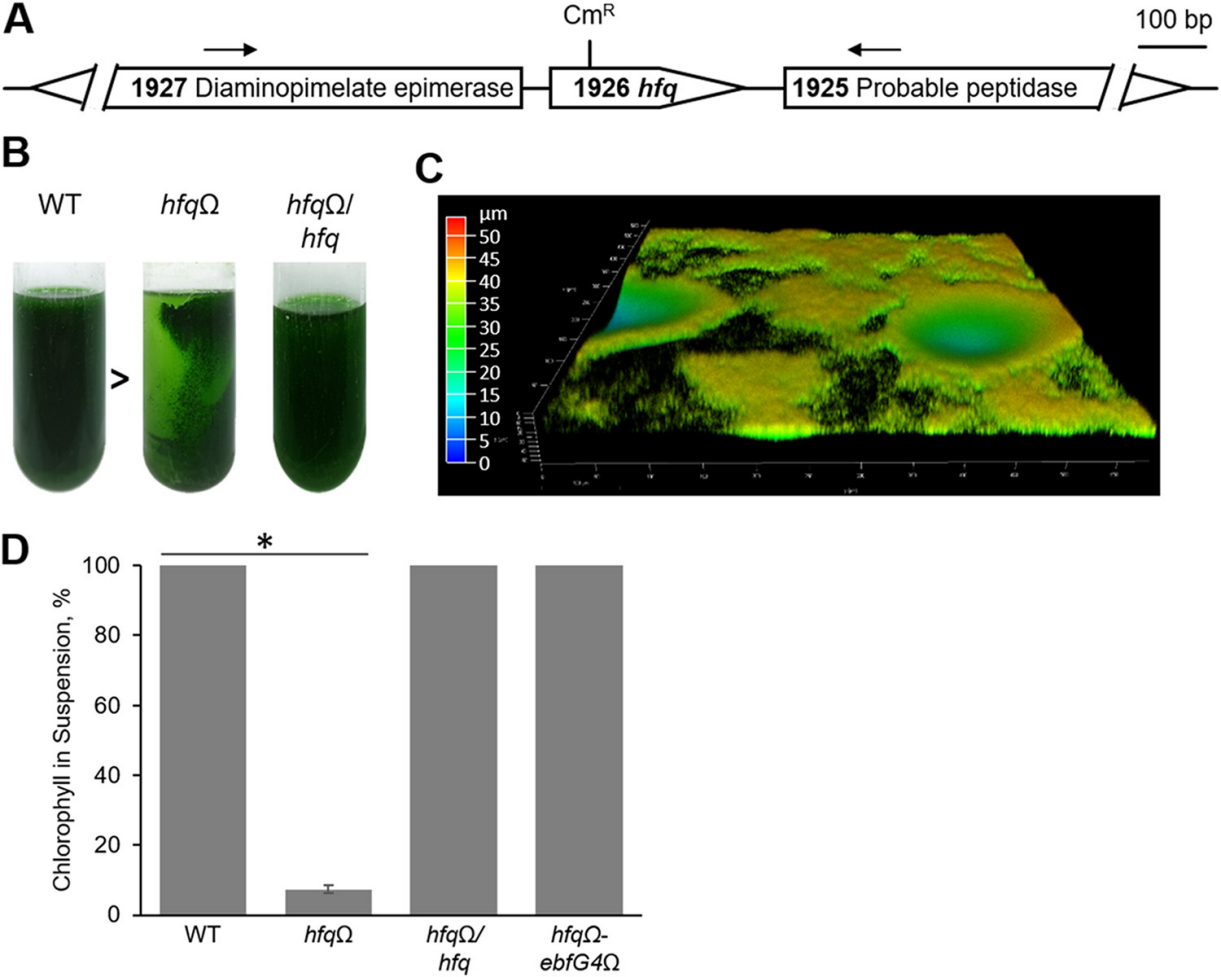
Hfq is required for biofilm self-suppression. (A) Map of the genomic region of Synpcc7942_1926. A chloramphenicol resistance fragment that is based on the omega cassette (Cm^R^) was used for gene inactivation to generate the *hfq*Ω mutant. Arrows indicate PCR primers used to amplify a DNA fragment that was introduced in *trans* into the *hfq*Ω mutant to generate the *hfq*Ω/*hfq* strain to test complementation. (B) Cultures of WT, *hfq*Ω, and *hfq*Ω/*hfq* strains. The arrowhead indicates the direction of light (biofilms are formed away from the light source). (C) Image of *hfq*Ω biofilm using confocal fluorescence microscopy. Imaging is based on autofluorescence (excitation at 630 nm and emission at 641 to 657 nm). The color scale represents biofilm depth. Different regions of the biofilm differ in depth (see Fig. S2 in the supplemental material). (D) Percentage of chlorophyll in suspension as a proxy for biofilm formation. Strains analyzed were the WT, the *hfq*Ω mutant, the *hfq*Ω/*hfq* strain, and the double mutant in which *hfq* and gene Synpcc7942_1134 are inactivated (*hfq*Ω *ebfG4*). Data represent averages and SD from 3 independent experiments. The asterisk indicates significance (*P* < 3.0E−20 by a two-tailed *t* test).

The presence of the Hfq RNA chaperone in a complex with two components, PilB and EbsA, that are both required for biofilm self-suppression in *S. elongatus* suggests that it is also involved in this mechanism. Indeed, the *hfq* knockout mutant (*hfq*Ω) resulting from the insertion of an omega cassette into Synpcc7942_1926 (Fig. 3A) develops robust biofilms, apparent as cells adhering to the growth tube (Fig. 3B). Additionally, fluorescence microscopy (Fig. 3C and Fig. S2) and measurements of relative chlorophyll in suspended cells (Fig. 3D, *hfq*Ω) indicated biofilm formation by the *hfq*Ω mutant. Furthermore, the insertion of Synpcc7942_1926 in *trans* into the *hfq*Ω mutant abolished biofilm development, confirming that Hfq is required for the biofilm self-inhibition process (Fig. 3D and Fig. S2; see also Fig. S4C for phenotypic complementation with FLAG-tagged Hfq). Also, similar to the *pilB*::Tn*5 ebfG4*Ω and *ebsA*::Tn*5 ebfG4*Ω mutants, the *hfq*Ω *ebfG4*Ω double mutant grew planktonically (Fig. 3D). Thus, the three mutants share a mechanism of biofilm development in which EbfG4 takes part.

### Impairment of EbsA or Hfq abrogates pilus formation.

**FIG 4:**
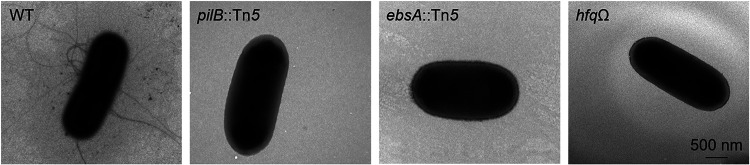
Transmission electron microscopy (TEM) images of negatively stained cells of the WT, *pilB*::Tn*5*, *ebsA*::Tn*5*, and *hfq*Ω strains. The scale bar is valid for all images.

The *pilB* mutant lacks cell pili (Fig. 4) (33), indicating that the ATPase homolog impaired in this strain has a role in pilus assembly. Given the physical association between PilB, EbsA, and Hfq (Fig. 2), we tested whether the latter two proteins take part in pilus assembly. Transmission electron microscopy (TEM) indicated that similar to the *pilB*::Tn*5* strain (33), the *ebsA*::Tn*5* and *hfq*Ω strains are completely devoid of cell pili, in contrast to the piliated WT strain (Fig. 4), thus demonstrating the requirement of EbsA and Hfq for pilus assembly. Moreover, this observation further supports the involvement of these proteins in the same cellular machinery as PilB, in agreement with the physical interaction among the proteins (Fig. 2). The involvement of Hfq in pilus assembly and its association with PilB were previously reported for the cyanobacterium *Synechocystis* sp. strain PCC 6803 (44, 46).

T4P systems are commonly involved in DNA transfer (47–51); accordingly, we noted previously that the *pilB*::Tn*5* mutant lost the natural ability of the WT strain to take up DNA (30). Therefore, we tested the transformability of the newly isolated mutants. The *pilB*::Tn*5* and *ebsA*::Tn*5* strains are absolutely nontransformable, whereas the transformation efficiency of the *hfq*Ω strain is about 100-fold lower than that of the WT (Fig. S5), in accordance with a recent publication (52).

10.1128/mBio.03674-20.5 FIG S5 DNA uptake by the WT, *pilB*::Tn*5*, *ebsA*::Tn*5*, and *hfq*Ω strains. (A) Table summarizing the number of CFU following plating onto nonselective and selective media. The transformation efficiency was calculated as the ratio of CFU obtained on selective versus nonselective medium for the particular strain. The efficiency observed for the WT is common for cultures grown under continuous light (A. Taton, C. Erikson, Y. Yang, B. E. Rubin, et al., Nat Commun 11:1688, 2020, https://doi.org/10.1038/s41467-020-15384-9). (B) Plating on selective medium. Images represent 1 μl (WT) or 200 μl (mutants) of the transformation mixture. A shuttle vector capable of replication in *S. elongatus* was used so that the transformation assay represents the ability to take up the DNA without the requirement for integration into the chromosome. Download FIG S5, TIF file, 2.8 MB. Copyright © 2021 Yegorov et al. 2021 Yegorov et al. https://creativecommons.org/licenses/by/4.0/ This content is distributed under the terms of the Creative Commons Attribution 4.0 International license.

### Impairment of EbsA or Hfq affects the exoproteome.

**FIG 5:**
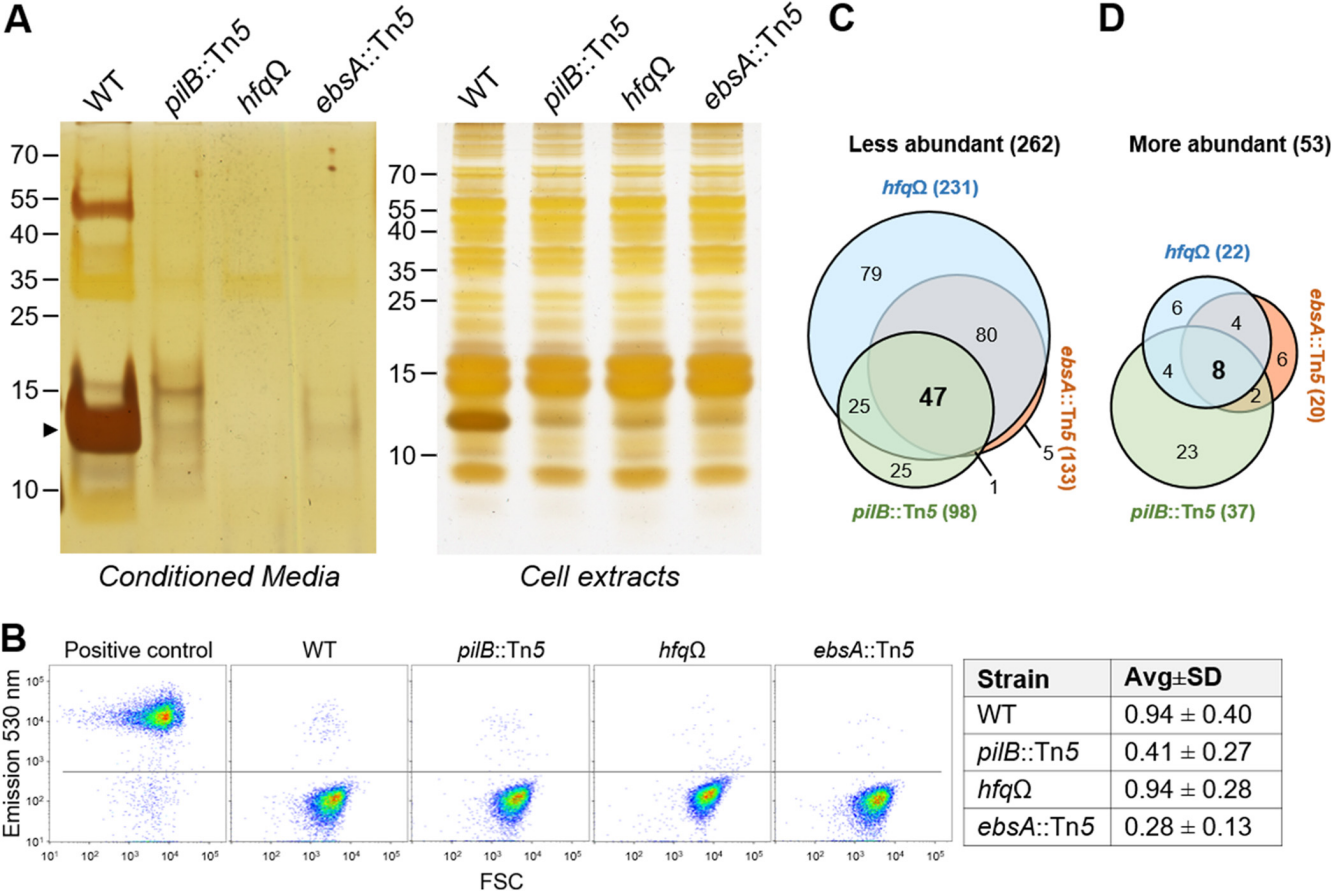
Exoproteome analyses of 2-day-old cultures of the WT, *pilB*::Tn*5*, *ebsA*::Tn*5*, and *hfq*Ω strains. (A) Electrophoresis followed by silver staining of concentrated conditioned media (CM) (left) or cellular extracts (right). Each lane represents 2 ml of culture fluids or 0.3 μg of chlorophyll (left and right, respectively). Numbers indicate molecular weight markers in kilodaltons. The arrowhead indicates the pilus subunit PilA. (B) Flow cytometry analyses following SYTOX live/dead staining. Density plots from a single representative experiment are presented (excitation at 488 nm and emission at 530 ± 25 nm). FSC, forward scattering. Heat-treated WT cells served as a positive control. Horizontal lines define the threshold for positive cells. The table summarizes percentages of SYTOX-positive cells (averages [Avg] ± SD) from three biological independent experiments. Differences between the cultures are insignificant (*P* > 0.09 by a two-tailed *t* test). (C and D) Venn diagrams presenting proteins less or more abundant in the exoproteomes. The number of proteins less abundant in the particular mutant (fold change of >2; FDR of <0.1) is indicated in parentheses. A total of 262 proteins are less abundant (C), 53 are more abundant (D), and only 6 proteins exhibited “opposite abundance” in different mutants; namely, they were more abundant in a particular mutant(s) but less abundant in another (see Data Set S3 in the supplemental material).

Components of the T4P assembly apparatus share homology with those of T2S systems (1, 7, 53–55). Furthermore, in particular cases, T4P machineries have been demonstrated to be involved in the secretion of proteins to the extracellular milieu (35). The inactivation of *pilB* affects the levels of extracellular proteins (here, the exoproteome) (33). Therefore, the association of EbsA and Hfq with PilB encouraged us to examine the exoproteomes of the *ebsA*::Tn*5* and *hfq*Ω strains. CM from planktonic (2-day-old) cultures of the WT, *pilB*::Tn*5*, *ebsA*::Tn*5*, and *hfq*Ω strains were concentrated by lyophilization and analyzed by electrophoresis and silver staining. This analysis indicated that several proteins are present in substantially reduced amounts or are absent from the exoproteome of the *pilB*::Tn*5* mutant compared to the WT (Fig. 5A, left), in agreement with previous studies (30, 33). Furthermore, lower levels of some proteins were observed in extracellular fluids from *ebsA*::Tn*5* and *hfq*Ω cultures than in *pilB*::Tn*5* cultures (Fig. 5A, left). The most prominent band in WT CM (∼13 kDa) (Fig. 5A, left, arrowhead), which corresponds to the pilin subunit PilA1, represents detached pili (33).

10.1128/mBio.03674-20.10 DATA SET S3 Proteins consistently less or more abundant in the mutant exoproteome(s) than in the WT exoproteome (fold change of less than −2.0 and FDR of <0.05 for less abundant proteins and fold change of >2.0 and FDR of <0.05 for more abundant proteins). Tab “More or less abundant” includes proteins with significant enrichment; however, these proteins were more abundant in a particular mutant(s) but less abundant in another(s). Data for all detected proteins from each exoproteome are supplied in the “Raw data” tab. Download Data Set S3, XLSX file, 0.3 MB. Copyright © 2021 Yegorov et al. 2021 Yegorov et al. https://creativecommons.org/licenses/by/4.0/ This content is distributed under the terms of the Creative Commons Attribution 4.0 International license.

Exoproteins are released to the extracellular milieu by regulated secretion processes; however, cell lysis may also contribute to the exoproteome. To test whether differences in cell lysis between the strains contribute to the observed changes in extracellular proteins, 2-day-old cultures (the growth stage at which the exoproteome was analyzed) were assessed by live/dead staining. SYTOX green nucleic acid stain enters cells with compromised membranes; accordingly, heat treatment of WT cells resulted in ∼96% positive cells (Fig. 5B, control). In cultures of the *pilB*::Tn*5*, *ebsA*::Tn*5*, and *hfq*Ω mutants, however, staining revealed only a minute fraction of positive cells (<1%), and differences among the strains were not significant (Fig. 5B). Thus, differential cell lysis does not contribute to the observed changes among the exoproteomes.

Additionally, quantitative analyses of extracellular fluids were performed using MS. The ratio of the abundance of a protein in CM of the WT versus that of a given mutant was used to identify proteins that are less abundant in the mutant exoproteome (Fig. 5C and Data Set S3) (fold change of >2; false discovery rate [FDR] of <0.1). As commonly done, samples were normalized based on the protein amount; namely, equal amounts of protein from each strain were digested by trypsin and subsequently analyzed by MS. Because the prominent ∼13 kDa protein (PilA) is missing from extracellular fluids of the mutants (Fig. 5A), equal total protein samples result in an exaggerated representation of the non-PilA protein complement in the exoproteome of the mutants. Thus, the normalization step underestimates the quantitative difference of proteins that are less abundant in the mutant exoproteomes. Nevertheless, MS analyses identified numerous exoproteins that are less abundant in the mutants, as specified below.

The inactivation of *pilB* decreased the complexity of the exoproteome (Fig. 5C), as previously reported (33); furthermore, many proteins are less abundant in the exoproteome of the *ebsA*::Tn*5* or *hfq*Ω mutant than in the WT (Fig. 5C). Of the 98 proteins that are less abundant in the *pilB*::Tn*5* exoproteome than in the WT, 47 (48%) are shared among the three mutants (Fig. 5C). This overlap is consistent with our suggestion that PilB, EbsA, and Hfq take part in the same protein secretion machinery, in agreement with co-IP data that indicate physical interactions among these proteins (Fig. 2). Numerous proteins, however, are less abundant in the *ebsA*::Tn*5* or *hfq*Ω mutant but not in the *pilB*::Tn*5* mutant (Fig. 5C), suggesting an additional effect of the inactivation of either *ebsA* or *hfq* on the exoproteome via a process that does not involve PilB.

Analysis of cell extracts was performed to examine whether the inactivation of either *pilB*, *ebsA*, or *hfq* substantially affects the overall amount of cellular proteins. This analysis revealed indistinguishable protein profiles with the exception of an ∼13 kDa protein that is much more intense in the WT than in any of the three mutants (Fig. 5A, right) and is likely to be PilA, in accordance with the absence of cell pili from the *pilB*::Tn*5*, *ebsA*::Tn*5*, and *hfq*Ω mutants (Fig. 4). Of note, 14 ribosomal subunit proteins were detected at low levels in the exoproteomes of the mutants, 4 of which are less abundant in the exoproteomes of all three mutants (Data Set S3, sheet “Less abundant—Ribosomal”). Ribosomal subunits are ubiquitous cellular components, and thus, drastic changes in their intracellular amounts should have been apparent by gel electrophoresis. For example, RplJ (175 amino acids [aa]) is ∼28-, ∼12-, and ∼7-fold less abundant in the *ebsA*::Tn*5*, *pilB*::Tn*5*, and *hfq*Ω mutants, respectively, than in the WT. The intensities of the bands in the corresponding regions of the total protein gel, however, are similar among the strains (Fig. 5A, right). Additionally, the levels of RpmE (77 aa) are ∼137- and ∼213-fold lower in the exoproteomes of the *ebsA*::Tn*5* and *hfq*Ω mutants, respectively; however, such changes are not observed in cellular extracts (Fig. 5A, right). Thus, the data suggest that the identified changes in the extracellular levels of ribosomal proteins do not merely represent variations in their cellular levels. Moreover, if changes in extracellular levels of ribosomal proteins are due to lower intracellular abundance, one would expect a substantial impact on protein synthesis and, consequently, reduced growth of the mutants, which was not observed (Fig. S3). Together, these data support the suggestion that at least some of the changes detected in the exoproteomes represent the involvement of EbsA and Hfq in secretion processes.

Analysis using MEME (45) revealed the enrichment of particular motifs in proteins that are less abundant specifically in the exoproteomes of the *ebsA*::Tn*5* and *hfq*Ω mutants (Table S2). A motif that includes two conserved cysteine residues (C-x-x-C), typical of thiol:disulfide oxidoreductases involved in redox reactions, is enriched (Table S2, *hfq*Ω). Additionally, the motif T-P-[STA]-P-[ST]-P is enriched in the less abundant proteins of the *hfq*Ω mutant and is part of a larger motif enriched in less abundant proteins of the *ebsA*::Tn*5* mutant. The function of these motifs, as well as that of the additional motif found in the *hfq*Ω sample (Table S2), is unknown. Enrichment analysis of less abundant proteins using STRING indicated the presence of signal secretion sequences in proteins less abundant in the *ebsA*::Tn*5* and *hfq*Ω exoproteomes than in the WT exoproteome (Data Set S2). This analysis did not reveal signal secretion sequences in proteins that were less abundant only in *pilB*::Tn*5* samples, in agreement with a previous comparative analysis of *pilB*::Tn*5* and WT exoproteomes (33).

10.1128/mBio.03674-20.7 TABLE S2 Analysis using MEME of proteins less abundant in exoproteomes of the *ebsA*::Tn*5*, *hfq*Ω, and *pilB*::Tn*5* strains than in the WT. MEME’s motif logo is indicated when the E value is <0.05. Motifs were significantly enriched in comparison to all proteome backgrounds (two-tailed *P* value of <0.05 by Fisher’s exact test except for motif 2 in the *ebsA*::Tn*5* mutant [*P* value of 0.0571]). *MEME analysis did not reveal a significant motif (E value of <0.05); however, a FIMO (Find Individual Motif Occurrences) search for the indicated motif patterns using the set of less abundant proteins of a particular mutant indicated individual motif occurrences. Download Table S2, PDF file, 0.6 MB. Copyright © 2021 Yegorov et al. 2021 Yegorov et al. https://creativecommons.org/licenses/by/4.0/ This content is distributed under the terms of the Creative Commons Attribution 4.0 International license.

In addition to the suggested abrogation of protein secretion, the inactivation of *pilB* resulted in the augmentation of particular proteins in the mutant’s exoproteome compared to the WT (Fig. 5D and Data Set S3), in agreement with our previous report (33). The “overabundant” proteins include some that enable biofilm development and carry a GG secretion motif (EbfGs). The eight proteins that are overabundant in the exoproteomes of all three mutants (Fig. 5D) include EbfG1, EbfG2, and EbfG4 (Data Set S3, sheet “More abundant in Mutants”). Note that on average, EbfG4 is 12-fold more abundant in *hfq*Ω than in WT samples (Data Set S3, sheet “Raw data”); however, due to the high variability between independent experiments, this difference is insignificant. The presence of EbfG proteins in the exoproteome correlates with biofilm development by the *pilB*::Tn*5* mutant (32); thus, the overabundance of EbfG proteins in the exoproteomes of the *ebsA*::Tn*5* and *hfq*Ω mutants is consistent with biofilm development by these mutants. Furthermore, the planktonic phenotype observed by the inactivation of *ebfG4* in the *pilB*::Tn*5* mutant (30) as well as in the *ebsA*::Tn*5* (Fig. 1D) and *hfq*Ω (Fig. 3D) mutants further supports a biofilm development process that involves the biofilm-enabling protein EbfG4 and that is common to the three mutant strains.

### Inactivation of the *Synechocystis* homolog of EbsA impairs protein secretion and motility.

**FIG 6:**
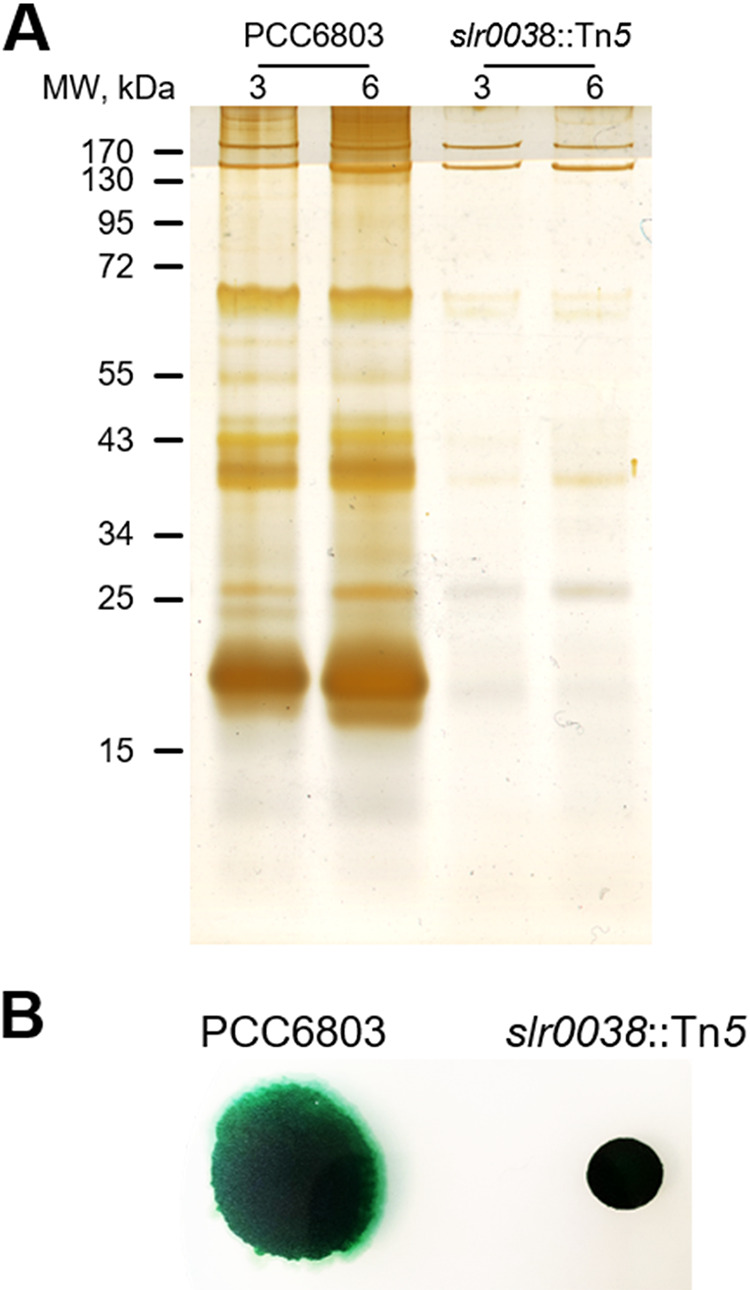
Inactivation of *slr0038* of *Synechocystis* PCC 6803 affects the exoproteome and motility. (A) Conditioned medium was harvested on days 3 and 6, and extracellular proteins were analyzed by gel electrophoresis followed by silver staining. MW, molecular weight. (B) Cultures were spotted onto agar plates. Cell spreading indicates motility. Strains analyzed were WT *Synechocystis* PCC 6803 (PCC6803) and the *slr0038*::Tn*5* mutant (inactivation of *slr0038*).

Homologs of EbsA are present in diverse cyanobacteria (Fig. S1). To examine whether the *Synechocystis* homolog serves similar functions, we inactivated the gene *slr0038*, encoding a protein that is 50% identical and 72% similar to EbsA. We suggest that the resulting mutant, *slr0038*::Tn*5*, is impaired in protein secretion, as revealed by protein electrophoresis followed by silver staining of concentrated CM (Fig. 6A).

*Synechocystis*, unlike the laboratory strain of *S. elongatus*, is motile on solid growth medium, and it has been shown that the abrogation of pilus formation impairs this motility (44, 46, 50, 51, 56). The lack of motility of the *slr0038* mutant (Fig. 6B) supports the suggestion that Slr0038 is required for pilus formation. Taken together, data from the analysis of extracellular proteins and motility support a role for Slr0038 in protein secretion and pilus assembly in *Synechocystis*, analogous to the role of EbsA in *S. elongatus*. The inactivation of *slr0038*, however, did not result in biofilm development, suggesting differences in the regulation of biofilm formation between these two cyanobacteria.

### Bioinformatics analysis suggests a single complex for secretion and T4P functions.

Studies of heterotrophic bacteria revealed the presence of distinct complexes for T2S or T4P biogenesis, the subunits of which are homologous (1, 7, 53–55). Recent analyses, however, indicate that distinct T2S complexes are largely confined to *Proteobacteria* (7, 34). Based on the observation that the inactivation of solely *pilB*, *ebsA*, or *hfq* affects the exoproteome (Fig. 5) as well as pilus assembly (Fig. 4), we propose that *S. elongatus* possesses a single complex that serves both protein secretion and pilus biogenesis, as was demonstrated for some cases of heterotrophic bacteria (35). Bioinformatic analysis of the *S. elongatus* genome, which revealed only a single gene for each component of a putative T2S/T4P system, supports the existence of a single complex, for example, Synpcc7942_2450 (annotated *gspD* [general secretion protein D]), Synpcc7942_2451 (annotated *pilO*), Synpcc7942_2452 (annotated *pilN*), Synpcc7942_2453 (annotated *pilM*), and Synpcc7942_2069 (annotated *pilC*). The absence of a second set of genes encoding putative components of an independent T2S, together with data indicating that the inactivation of a single gene impairs pilus assembly and affects the exoproteome, led us to propose that a single complex serves overlapping T2S and T4P functions in *S. elongatus*. Likewise, bioinformatics analysis and the phenotype obtained from the inactivation of *slr0038*, encoding an EbsA homolog in *Synechocystis*, support the existence of such a shared complex in other cyanobacteria.

## DISCUSSION

Several lines of evidence support the suggestion that two newly identified components, EbsA and Hfq, act along with PilB to affect biofilm formation via a common mechanism: (i) genetic evidence demonstrating the requirement of *ebfG4* for biofilm formation by the *pilB*::Tn*5*, *ebsA*::Tn*5*, and *hfq*Ω mutants (Fig. 1D and Fig. 3D); (ii) analysis of the exoproteome demonstrating high levels of biofilm-enabling proteins, including EbfG4, in CM from mutants (see Data Set S3 in the supplemental material); and (iii) the physical interaction among PilB, EbsA, and Hfq (Fig. 2). Nevertheless, the possibility that EbsA and Hfq also exert biofilm regulation independently of PilB should not be excluded.

### A tripartite complex, PilB-EbsA-Hfq, of a shared pilus assembly and protein secretion machinery.

**FIG 7:**
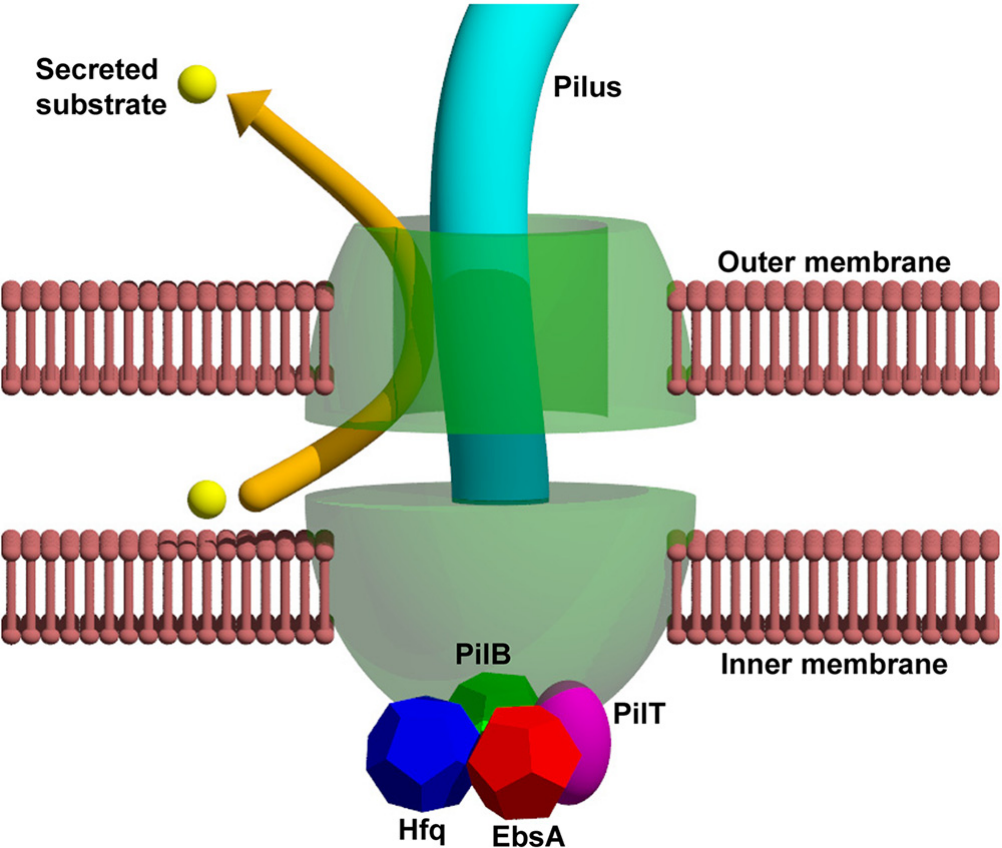
Proposed model for involvement of the tripartite complex PilB-EbsA-Hfq in a shared machinery for pilus assembly and protein secretion. The T4P/secretion complex is schematically depicted, showing only the inner and outer membrane components PilC and PilQ, respectively.

Based on this study we suggest that the tripartite complex PilB-EbsA-Hfq is associated with machinery involved in several cellular functions: pilus assembly (Fig. 4), DNA uptake (Fig. S5), and protein secretion (Fig. 5). Although it is well established that numerous bacteria possess distinct complexes for these functions (1, 7, 53–55), the T4P complexes in some bacteria are known to serve for the secretion of proteins other than the pilus subunits (35). For example, in Vibrio cholerae (57) and Dichelobacter nodosus (58), T4P systems are used for the secretion of a soluble colonization factor and a protease, respectively. Additionally, T4P-mediated secretion was demonstrated in Escherichia coli (59) and Francisella tularensis (60). The physical association of EbsA and Hfq with PilB, and the shared phenotypes of their mutants, strongly supports the suggestion that *S. elongatus* employs the same complex for pilus biogenesis and protein secretion (Fig. 7).

EbsA, a previously unknown protein that may be a component of T4P/secretion systems, is widespread in diverse cyanobacteria, and the inactivation of the *Synechocystis* homolog, *slr0038*, impairs protein secretion and, based on the lack of motility on agar, pilus function (Fig. 6). Similar to the *ebsA*::Tn*5* strain, the inactivation of *hfq* in *Synechocystis* blocks pilus formation and motility on agar plates (44). The involvement of *Synechocystis* Hfq in pilus formation, in analogy to its role in *S. elongatus*, together with the phenotype of the *slr0038*::Tn*5* strain, further substantiates the presence of a mechanism that is common to divergent cyanobacteria.

Exoproteome analyses imply pleiotropic effects of the inactivation of *ebsA* or *hfq*. About half of the proteins that are less abundant in the *pilB*::Tn*5* mutant (47 of 98) are also less abundant in the *ebsA*::Tn*5* and *hfq*Ω mutants. However, a larger number of proteins are less abundant than in the WT for the *ebsA*::Tn*5* and *hfq*Ω mutants compared to the *pilB*::Tn*5* mutant. Furthermore, a data set of less abundant *ebsA*::Tn*5* proteins is almost entirely encompassed by that group from the *hfq*Ω mutant. We propose that EbsA and Hfq also act in a secretion process that is independent of PilB, in agreement with enrichment analysis using STRING that showed secretion signal sequences in proteins that are less abundant in the *ebsA*::Tn*5* and *hfq*Ω exoproteomes than in the WT exoproteome, which was not true for the less abundant proteins of the *pilB* mutant. The 79 proteins that are less abundant specifically in the *hfq*Ω mutant (Fig. 5C) may represent an indirect effect of the Hfq RNA chaperone on protein expression or secretion. Numerous studies of Hfq in heterotrophic bacteria revealed its involvement in the regulation of translation (40–43). A role for cyanobacterial Hfq as an RNA chaperone has not been demonstrated yet; however, it is possible that it exerts translation regulation and that, consequently, the less abundant proteins unique to the *hfq*Ω mutant represent the outcome of the impact of Hfq on translation. Less abundant exoproteins of the *ebsA*::Tn*5* mutant, which are not shared with the *pilB*::Tn*5* mutant, may also represent a pleiotropic effect, although the particular mechanism in this case is enigmatic.

Some of the proteins that are underabundant in the mutants’ exoproteomes are cellular proteins, e.g., ribosomal subunits and a nucleoid protein (Data Set S3). Intracellular proteins that serve another function when present extracellularly, so-called “moonlighting proteins,” have been described in diverse organisms, including bacteria (61, 62). Such protein multitasking, however, has not been documented in cyanobacteria, and the reason for the release of *S. elongatus* cellular proteins is unknown. However, our data argue against cell lysis or general permeability changes as an explanation for these factors outside the cell.

In summary, this study is consistent with a hypothesis assigning a secretion role to the complex that assembles pili in cyanobacteria, as has been shown for a few species of *Proteobacteria*, and also identified distinct features of the cyanobacterial complex. Cyanobacterial cells possess an elaborate internal network of photosynthetic membranes in addition to their inner and outer cell membranes. Such morphology imposes further challenges for protein sorting and delivery, compared to heterotrophic bacteria. Conceivably, cyanobacteria evolved to accommodate these challenges by recruiting EbsA and Hfq to the T4P/secretion machinery.

## MATERIALS AND METHODS

### Strains, culture conditions, biofilm quantification, and assessment of DNA competency.

The growth of all Synechococcus elongatus PCC 7942 and *Synechocystis* PCC 6803 strains was performed in BG-11 medium as described previously (39). Quantification of biofilms based on chlorophyll measurements was performed as described previously (39). Briefly, suspended cells were carefully removed, and chlorophyll from the biofilm cells remaining in the growth tube was extracted with acetone. Additionally, acetone extraction was used to quantify chlorophyll in the suspended fraction. The percentage of chlorophyll in suspended cells from the total culture chlorophyll served for quantitative assessment of biofilms. Assessment of DNA competence was performed essentially as described previously (63). Exponentially growing cells were centrifuged (5,000 × *g* for 8 min at room temperature), washed once with 10 mM NaCl, and resuspended to an optical density at 750 nm (OD_750_) of 4.0. A shuttle vector (1,000 ng) was added to 600 μl of cells, which were gently agitated overnight at 28°C in the dark. Transformants were selected by plating on selective solid growth medium (50 μg/ml spectinomycin) supplemented with NaHCO_3_ (5 mM) and sodium thiosulfate (0.3%, wt/vol). Additionally, serial dilutions were plated onto nonselective medium. The transformation efficiency was calculated as the number of CFU per milliliter obtained on selective medium normalized to that observed on nonselective medium. The shuttle vector replicates autonomously, thus allowing the assessment of DNA uptake without a possible impact of the efficiency of DNA integration into the chromosome.

### Microscopy.

Examination of negatively stained cells by transmission electron microscopy was conducted at the Irving and Cherna Moskowitz Center for Nano and Bio-Nano Imaging at the Weizmann Institute of Science as described previously (33).

To observe biofilms by fluorescence microscopy, a sterile microscope slide was inserted into a growth tube upon culture inoculation, and biofilms formed on the glass tube wall as well as on the microscope slide. Following 7 days of growth, the microscope slide was removed with forceps and washed once by dipping into double-distilled water. Autofluorescence-based images were collected using a Leica SP8 confocal microscope (excitation at 630 nm and emission at 641 to 657 nm).

### IP followed by MS.

IP was performed essentially as described previously (44), with the following modifications. A culture (250 ml; OD_750_ of 2.5 to 3) was concentrated by centrifugation to 2.2 ml, and a freshly prepared protease inhibitor cocktail (catalog number P8465-5ML; Sigma) was added to 3.44 mg/ml. Aliquots (1 ml) of the concentrated cell culture were combined with ∼1 g of glass beads (catalog number 11079101; Biospec) in 2-ml Eppendorf tubes. Cells were broken using a mixer mill (catalog number MM400; Retch) at a frequency of 30 s^−1^ for 2 min in prechilled holders (5 times, with 1 min of incubation on ice between the cycles). Cell lysates were centrifuged (relative centrifugal force [RCF] of 835 × *g* for 5 min at 4°C) to pellet the glass beads. The cell lysate (1 ml) was transferred to a 1.5-ml Eppendorf tube containing 100 μl washed anti-FLAG magnetic beads (catalog number M8823; Sigma) and incubated for 2 h at room temperature and overnight at 4°C with mixing (RotoFlex; Argos Technologies). Beads were washed 4 to 5 times according to the manufacturer’s instructions, and elution was performed with 1 ml of 100 μM triple-FLAG peptide (catalog number A6001; APExBIO).

MS analysis at the de Botton Institute for Protein Profiling at The Nancy and Stephen Grand Israel National Center for Personalized Medicine (Weizmann Institute of Science) was performed as previously described (32), except that trypsin digestion was not followed by chymotrypsin digestion and “discovery mode” was used rather than “targeted analysis.” Raw data were processed with MaxQuant v1.6.0.16. The data were searched with the Andromeda search engine against the cyanobacterial proteome database appended with common laboratory protein contaminants. Quantification was based on the label-free quantitative (LFQ) method. Data were analyzed to identify proteins that were significantly enriched in a “bait strain” (triple-FLAG-tagged protein) compared to its nontagged negative control (*P* value of ≤0.05 by a *t* test; fold change of >6).

### Analysis of extracellular fluids and total cell extracts.

For collection of CM, cultures were centrifuged (5,000 × *g* for 10 min) at room temperature, and the supernatant was removed and passed through a 0.22-μm filter. MS analysis was performed as described above. Data were analyzed to identify proteins that are significantly more or less abundant in a particular mutant’s exoproteome than in the WT (FDR of ≤0.1), with a cutoff of at least a 2-fold change. Protein motifs were searched using the MEME package (45) (parameters -nmotifs 10, -minw 6, -maxw 50, and -mod anr). Significant relevant motifs were scanned against the *S. elongatus* proteome using Find Individual Motif Occurrences (FIMO) (45) to reveal motif occurrences in the total proteome. Enrichment was calculated for each significant motif by comparing its occurrence in a specific protein list with its FIMO background output data (two-tailed *P* value of <0.05 by Fisher’s exact test).

Additionally, exoproteins as well as cell extracts were analyzed by using Tricine gel (64) followed by silver staining (SilverSNAP stain kit II, catalog number 24612; Pierce). For this analysis, CM collected as described above was lyophilized and resuspended in 1/200 of the original volume in a solution containing 10 mM Tris (pH 8.0) and 1 mM EDTA supplemented with 0.86 mg/ml of a freshly prepared protease inhibitor cocktail (catalog number P8465-5ML; Sigma). Cell extracts were prepared essentially as described above for the IP analysis; however, a 50-ml culture was concentrated to 0.5 ml, the protease inhibitor concentration was 0.86 mg/ml, and 0.2 g of glass beads was added for breakage by the mixer mill (catalog number MM400; Retch).

### Live/dead staining.

SYTOX green dead cell stain (Molecular Probes), a dye that enters cells with compromised membranes and binds to nucleic acids, was employed for viability assessment as described previously (65). For SYTOX staining, cells were diluted with phosphate saline buffer to an OD_750_ of 0.0004. Following the addition of SYTOX (40 nM), cells were incubated in the dark for 15 min and analyzed by flow cytometry using Becton, Dickinson Fortessa (excitation at 488 nm and emission at 530 ± 25 nm). Heat-treated (80°C for 1 min) WT cells served as positive controls. Fluorescence was plotted versus forward scattering (FSC).
