# Supplementary figures and images for: A Cyanobacterial Component Required for Pilus Biogenesis Affects the Exoproteome
Source: mBio. 2021 Mar 16;12(2):e03674-20. doi: 10.1128/mBio.03674-20 (PMC8092324; doi:10.1128/mBio.03674-20)

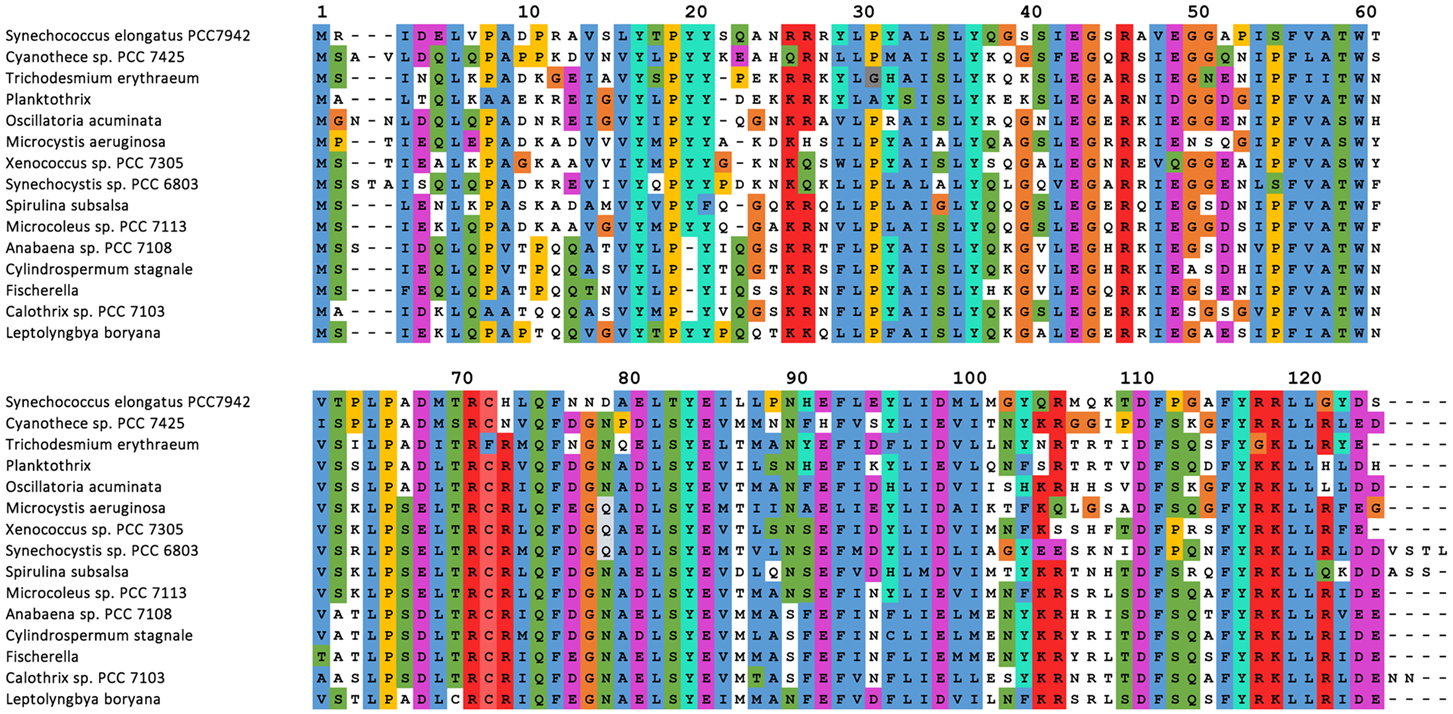

Supplement: FIG S1 [file mBio.03674-20-sf001.tif]

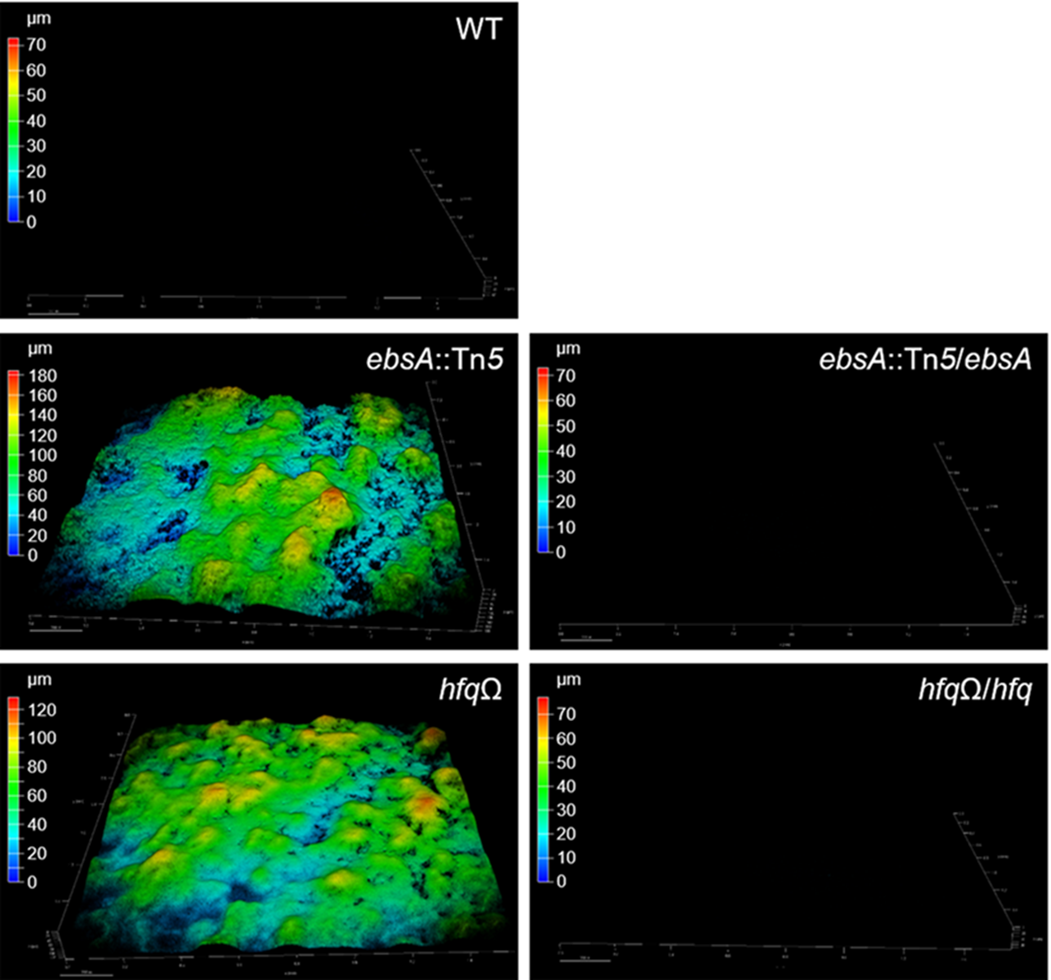

Supplement: FIG S2 [file mBio.03674-20-sf002.tif]

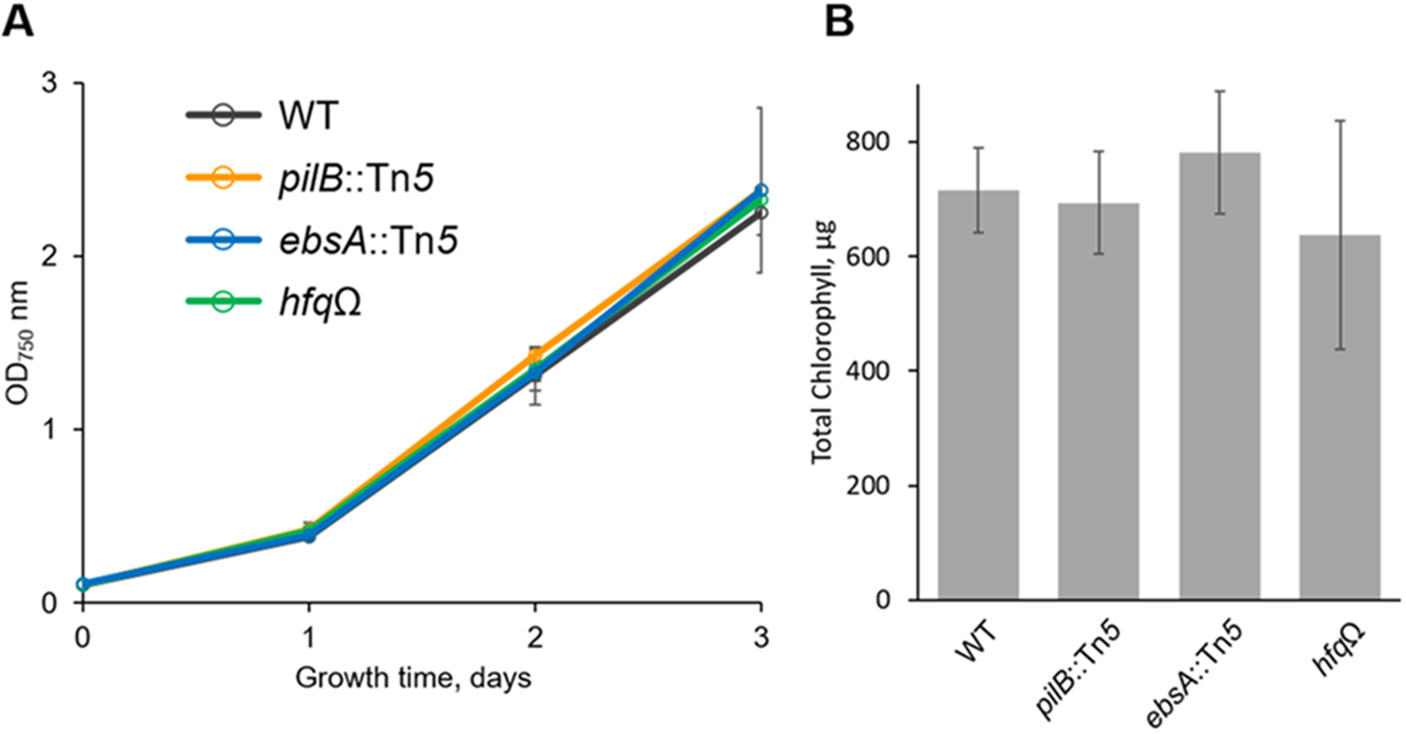

Supplement: FIG S3 [file mBio.03674-20-sf003.tif]

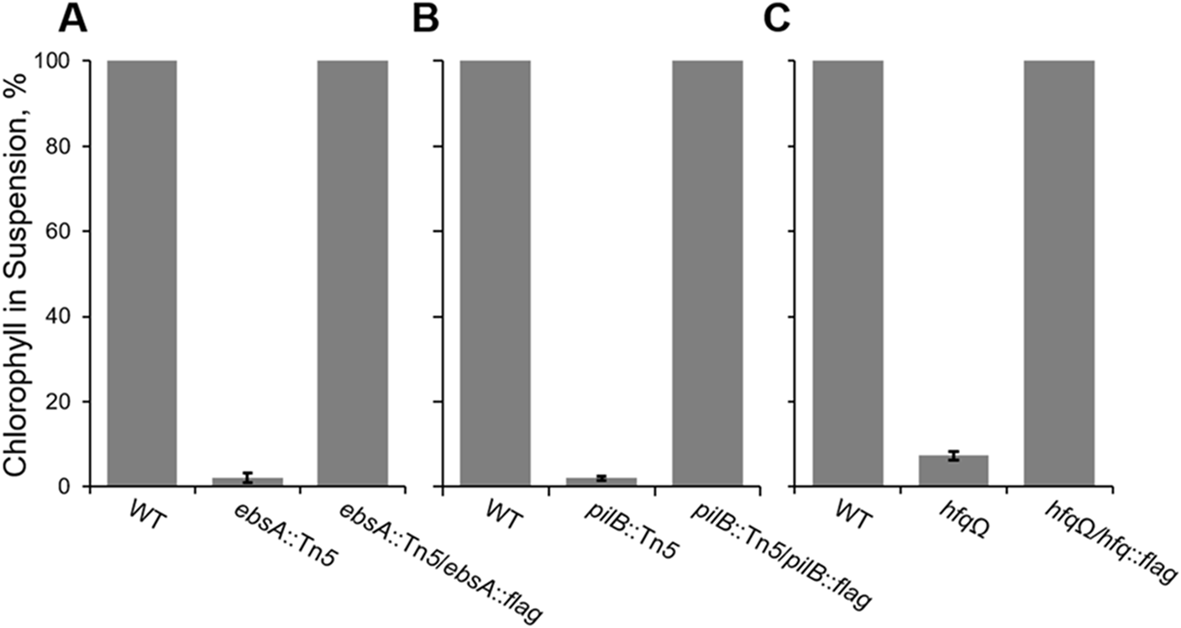

Supplement: FIG S4 [file mBio.03674-20-sf004.tif]

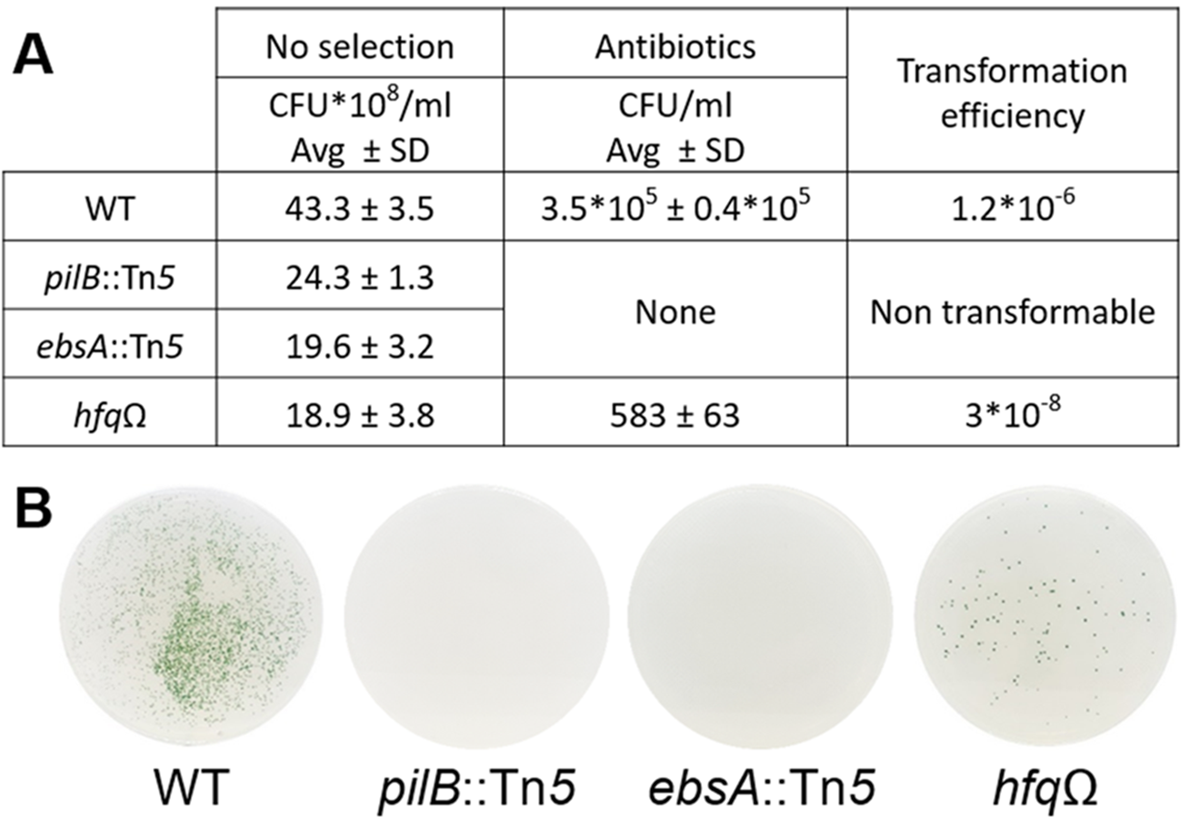

Supplement: FIG S5 [file mBio.03674-20-sf005.tif]
