# Supplementary material for: A Cyanobacterial Component Required for Pilus Biogenesis Affects the Exoproteome
Source: mBio. 2021 Mar 16;12(2):e03674-20. doi: 10.1128/mBio.03674-20 (PMC8092324; doi:10.1128/mBio.03674-20)
Supplement: TABLE S1 [file mBio.03674-20-st001.pdf]

| **Gene disruption** | **Primer sequence (upper forward, lower reverse)** | **Insertion of antibiotic cassette** |
|---|---|---|
| Inactivation of *hfq** (Synpcc7942_1926) | CATGCTGACGGTTGTGACC<br>CATCAACACGATCGAACTCG | *Nhe*I, $Cm^R$ |
| Inactivation of *slr0038** | GTCAACAATGCCCAGAGC<br>AGATTGCCTGGACCTTCC | *Dra*II, $Km^R$ |
| **Gene disruption using transposon insertion vector** | | |
| Inactivation of *ebsA*** (Synpcc7942_0862) | UGS vector 22D7 | 18 bp from the start codon, $Km^R$ |

| **Cloning of fragments encoding native and FLAG-tagged proteins** | | |
|---|---|---|
| | **Primer sequence (upper forward, lower reverse)** | **Purpose** |
| *hfq* comp# | CATGCTGACGGTTGTGACC<br>AGGACTGGATGCTGACTTGC | PCR amplification |
| *hfq*::3xflag# | CATGCTGACGGTTGTGACC<br>ctacttatcgtcgtcatccttgtaatcgatatcgtgatccttgtaatccccatc gtgatccttgtaatcGAGGAGGGGTCGCAGCGA | PCR amplification |
| *ebsA* comp# | AAGCAACCGAGAAACGGCAG<br>CAGCGTCAGTGCTGACTTAC | PCR amplification |
| *ebsA*::3xflag# | AAGCAACCGAGAAACGGCAG<br>ttacttatcgtcgtcatccttgtaatcgatatcgtgatccttgtaatccccatc gtgatccttgtaatcGCTGTCGTAACCCAGCAATC | PCR amplification |
| *pilB* comp# | GACGATTGAGAGTCCTACC<br>TTAGTTCCCAAAGCGGGGCGT | PCR amplification |
| *pilB*::3xflag# | GACGATTGAGAGTCCTACC<br>ttacttatcgtcgtcatccttgtaatcgatatcgtgatccttgtaatccccatc gtgatccttgtaatcGTTCCCAAAGCGGGGCGT | PCR amplification |

| Additional primers | | Purpose |
|---|---|---|
| pJet F/R | CGACTCACTATAGGGAGAGCGGC | Sequencing |
| | AAGAACATCGATTTTCCATGGCAG | |
| Shuttle Vector | TAATACGACTCACTATAGGG | Sequencing |
| | CAGGAAACAGCTATGAC | |
| *pilB* ins F/R | AATGATCCGGCGATCAATATC | Examine segregation |
| | GTAGGGCTCCACTCCCATT | |
| NS 1 F/R | CGTCGAAGATGGAAAAGCTC | Examine segregation |
| | ATTGACCCGGTAGGGATTTC | |
| NS 2 F/R | GGCAGGATTGCGATCGAGTT | Examine segregation |

**Table S1: Summary of cloning information**

* Gene inactivation vector was initially constructed by cloning a PCR product amplified with the indicated primers into pJET1.2. Subsequently, the specified restriction site was used for insertion of either chloramphenicol resistance cassette ($Cm^R$) that is based on the Ω inactivation fragment (P. Prentki and H. M. Krisch, Gene 29:303-313, 1984) or kanamycin resistance cassette ($Km^R$) from transposon Tn*5* (E. Beck, G. Ludwig, E. A. Auerswald, B. Reiss and H. Schaller, Gene 19:327-336, 1982), as indicated. Accordingly, the mutants are denoted *hfq*Ω and *ebsA*::Tn*5*.

** Transposon insertional inactivation was performed using the indicated vector from the unigene set (UGS) library (C.K. Holtman, Y. Chen, P. Sandoval, A. Gonzales, M.S. Nalty, T.L. Thomas, P. Youderian, and S.S. Golden, High-throughput functional analysis of the *Synechococcus elongatus* PCC 7942 genome. DNA research: an international journal for rapid publication of reports on genes and genomes 12:103-115, 2005). Gene disruption in *S. elongatus* was obtained by transformation and replacement of the native gene by homologous recombination.

# PCR products were cloned into the *Swa*I in NSII or NSI (*hfq* and *pilB*, respectively) or *Eco*RV in shuttle vector pSZ2 (*ebsA*).

All cloning products were validated by PCR analyses and sequencing. Complete chromosome segregation of cyanobacterial strains were confirmed by PCR.

Lowercase letters in primers indicate the regions that encode the triple FLAG-tag.

** Transposon insertional inactivation was performed using the indicated vector from the unigene set (UGS) library (3). Gene disruption in *S. elongatus* was obtained by transformation and replacement of the native gene by homologous recombination.

# PCR products were cloned into the *Swa*I in NSII or NSI (*hfq* and *pilB*, respectively) or *Eco*RV in shuttle vector pSZ2 (*ebsA*).

All cloning products were validated by PCR analyses and sequencing. Complete chromosome segregation of cyanobacterial strains were confirmed by PCR.

Lowercase letters in primers indicate the regions that encode the triple FLAG-tag.

**REFERENCES**

1. Prentki P, Krisch HM. 1984. In vitro insertional mutagenesis with a selectable DNA fragment. Gene 29:303-13.
2. Beck E, Ludwig G, Auerswald EA, Reiss B, Schaller H. 1982. Nucleotide sequence and exact localization of the neomycin phosphotransferase gene from transposon Tn*5*. Gene 19:327-36.
3. Holtman CK, Chen Y, Sandoval P, Gonzales A, Nalty MS, Thomas TL, Youderian P, Golden SS. 2005. High-throughput functional analysis of the *Synechococcus elongatus* PCC 7942 genome. DNA Res 12:103-15.
