# Supplementary material for: A Cyanobacterial Component Required for Pilus Biogenesis Affects the Exoproteome
Source: mBio. 2021 Mar 16;12(2):e03674-20. doi: 10.1128/mBio.03674-20 (PMC8092324; doi:10.1128/mBio.03674-20)
Supplement: TABLE S2 [file mBio.03674-20-st002.pdf]

	Motif pattern	***ebsA*::Tn*5***	***hfq*Ω**	***pilB*::Tn*5***
**1**	C-x-x-C	*Appears in 9 proteins	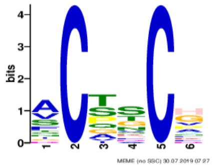 Appears in 20 proteins	*Appears in 7 proteins
**2**	T-P-[STA]-P-[ST]-P	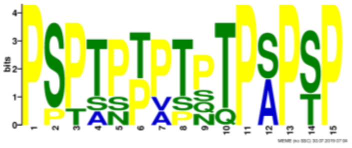 Appears in 4 proteins	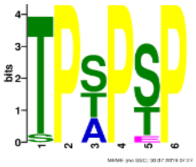 Appears in 14 proteins	*Appears in 6 proteins
**3**	G-[GN]-x-[GN]-[NA]-D-[TS]-I	*Appears in 4 proteins	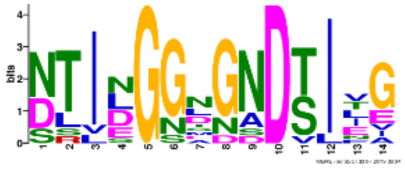 Appears in 12 proteins	*Appears in 2 proteins

Table S2: Analysis using MEME of proteins less abundant in exo-proteomes of *ebsA*::Tn*5*, *hfq*Ω, and *pilB*::Tn*5* compared to WT.

MEME's motif logo is indicated when E-value<0.05. Motifs were significantly enriched in comparison to all proteome background (Fisher exact test, two tailed p-value < 0.05 except for motif 2 in *ebsA*::Tn*5*; p-value = 0.0571).

* MEME analysis did not reveal significant motif (E-value < 0.05), however, FIMO (Find Individual Motif Occurrences) search for the indicated motif patterns using the set of less abundant proteins of a particular mutant indicated individual motif occurrences.
